# Hydroxychloroquine-induced hyperpigmentation of the skin and bull’s-eye maculopathy in rheumatic patients: a case report and literature review

**DOI:** 10.3389/fimmu.2024.1383343

**Published:** 2024-04-10

**Authors:** Ji-peng Peng, Xiao-yu Yang, Feng Luo, Xue-mei Yuan, Hong Xiong, Wu-kai Ma, Xue-ming Yao

**Affiliations:** ^1^ Guizhou University of Traditional Chinese Medicine, Guiyang, China; ^2^ Department of Rheumatology and Immunology, Second Affiliated Hospital of Guizhou University of Traditional Chinese Medicine, Guiyang, China

**Keywords:** hydroxychloroquine, adverse drug reaction, hyperpigmentation of the skin, bull’s-eye maculopathy, literature review, autoimmune disease

## Abstract

Hydroxychloroquine (HCQ) is used as a traditional disease-modifying antirheumatic drugs (DMARDs), for the treatment of autoimmune diseases such as rheumatoid arthritis (RA) and systemic lupus erythematosus (SLE). However, it can cause serious adverse reactions, including hyperpigmentation of the skin and bull’s-eye macular lesions. Here, we present a case of HCQ-induced hyperpigmentation of the skin and bull’s-eye macular lesions in a patient who received HCQ for RA. A 65-year-old female patient developed blurred vision and hyperpigmentation of multiple areas of skin over the body for one month after 3 years of HCQ treatment for RA. Based on clinical presentation, ophthalmological examination and dermatopathological biopsy, a diagnosis of drug-induced cutaneous hyperpigmentation and bullous maculopathy of the right eye was made. After discontinuation of HCQ and treatment with iguratimod tablets, the hyperpigmentation of the patient ‘s skin was gradually reduced, and the symptoms of blurred vision were not significantly improved. We also reviewed the available literature on HCQ-induced cutaneous hyperpigmentation and bull’s-eye macular lesions and described the clinical features of HCQ-induced cutaneous hyperpigmentation and bull’s-eye macular lesions. In conclusion, clinicians should be aware of early cutaneous symptoms and HCQ-associated ophthalmotoxicity in patients with rheumatic diseases on HCQ sulphate and should actively monitor patients, have them undergo regular ophthalmological examinations and give appropriate treatment to prevent exacerbation of symptoms.

## Introduction

Hydroxychloroquine (HCQ) is a 4-aminoquinoline antimalarial drug. It is commonly used as a sulfate, namely HCQ sulfate. Its antimalarial effect is the same as that of chloroquine, but its toxicity is only half that of chloroquine. In addition, HCQ sulfate also has anti-inflammatory, immunomodulatory and anticoagulant effects (1–4), so it is widely used in clinical treatment of rheumatoid arthritis (RA), systemic lupus erythematosus (SLE), Sjögren’s syndrome, skin diseases, etc., and the adverse reactions are gradually increasing (5, 6). There are fewer reports on HCQ-induced hyperpigmentation of the skin and macular lesions in the bull’s eye. This article analyses the literature related to HCQ sulfate-induced hyperpigmentation of the skin and macular lesions in the bull’s eye in conjunction with the literature review by taking a case of HCQ sulfate-induced hyperpigmentation and macular lesions in the bull’s eye as an example in order to warn the clinic to fully understand the adverse effects of HCQ.

## Case report

**Figure 1:**
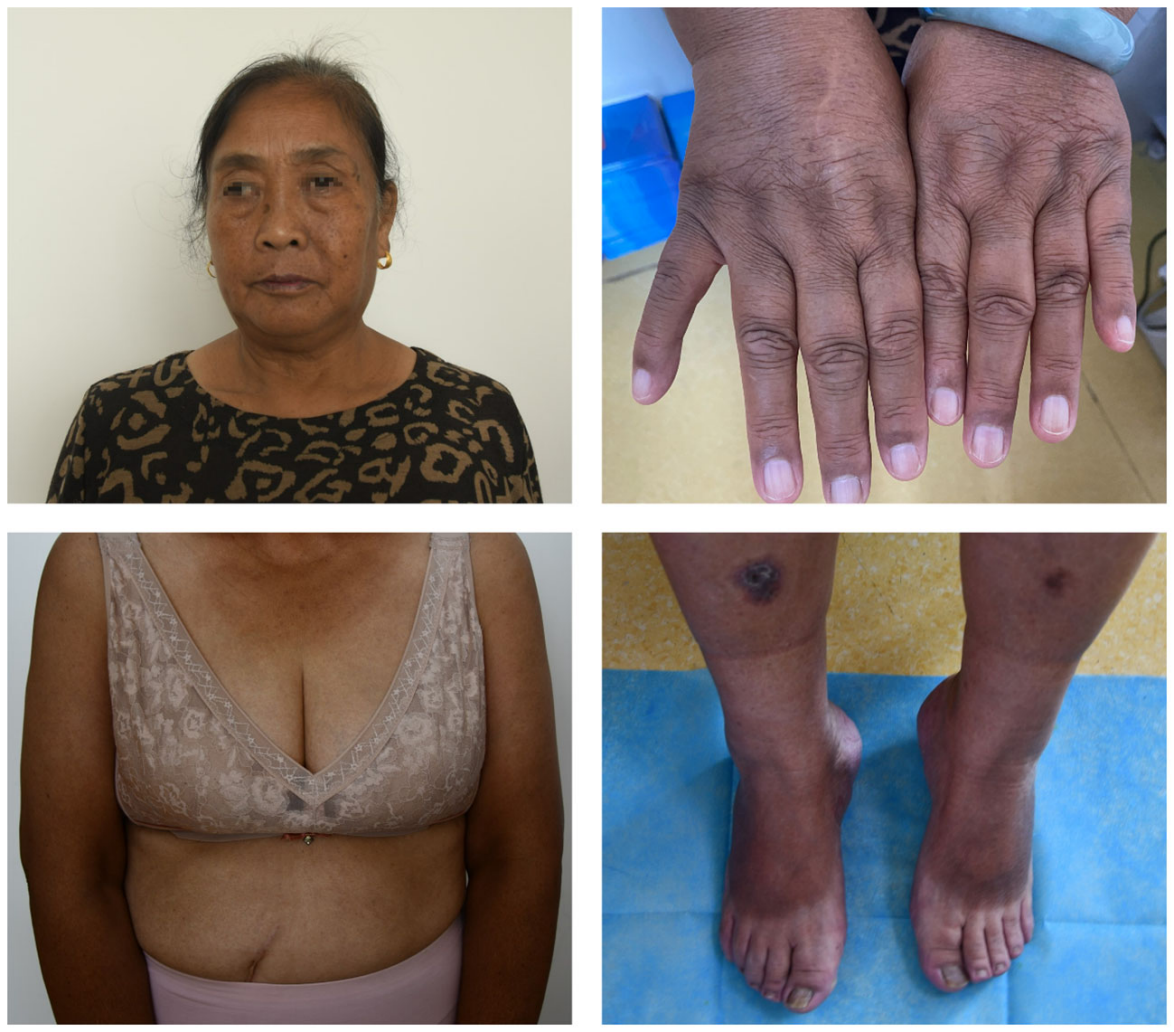
Hyperpigmentation of head, face, neck, upper limbs and lower limbs.

We assessed a 65-year-old female patient with RA in December 2023 who had been diagnosed with RA 10 years earlier and was now feeling pain in both shoulders, wrists, and finger joints of both hands with mild limitation of movement. The patient was treated with HCQ (400mg/d) and low-dose prednisolone (5 mg/d) for 3 years. The patient had no other comorbidities or medications, and for the past month felt blurred vision and noticed a darkening of the skin color. Our physical examination revealed excessive skin pigmentation in many parts of the body, especially in the head and face, neck, upper limbs and lower limbs (**Figure 1**). The patient attributed this to chronic ultraviolet light exposure.

**Figure 2:**
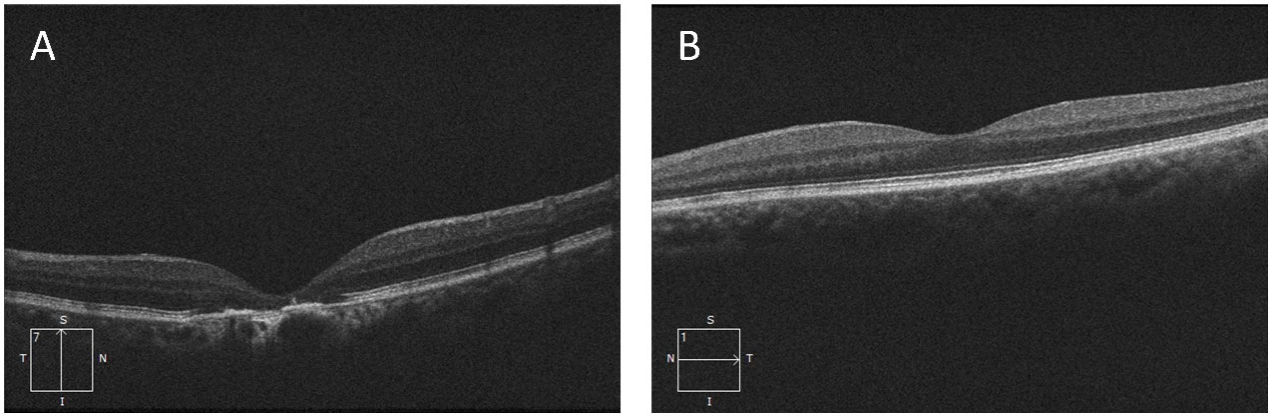
**(A)** OCT results of the right eye; **(B) ** OCT results of the left eye.

**Figure 3:**
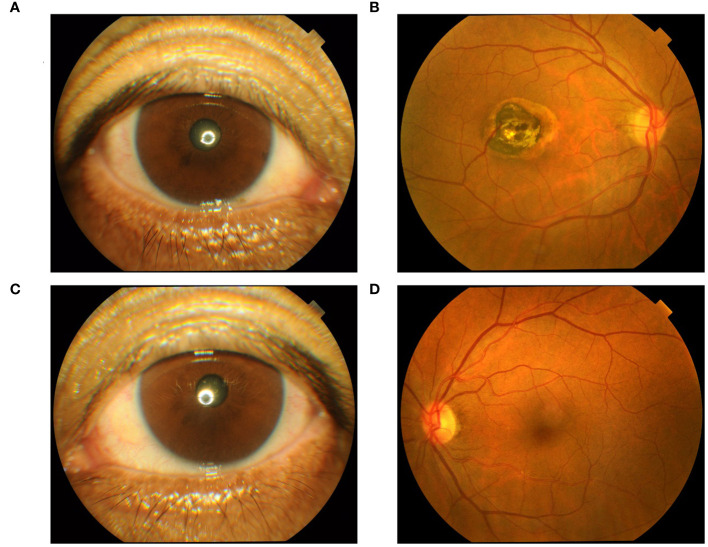
**(A) ** Right anterior segment findings; **(B)** Right eye fundus screening results; **(C)** Left anterior segment findings; **(D)** Left eye fundus screening results.

We performed relevant laboratory tests on the patient, which showed an elevated erythrocyte sedimentation rate of 61.00 mm/h (normal, 0-20 mm/L), an elevated rheumatoid factor of 43.15 IL/ml (normal, 0-20 IL/ml), and an elevated anti-cyclic citrullinated peptide antibody of > 500.00 U/mL (normal, 0-20 U/mL). We performed an ophthalmological examination of the patient because he had symptoms of blurred vision and because studies have shown that HCQ can cause retinopathy (7, 8). The visual field examination results showed that the visual field of the right eye showed a visual field defect outside the range of 45° above and 25° on the nasal side, and the light sensitivity of the remaining visual field decreased significantly. Compared with the dark spots around the physiological blind spots of the left eye, the photosensitivity decreased widely outside the range of 40°, and the photosensitivity decreased scattered within the range of 40°. Therefore, we performed optical coherence tomography (OCT) (**Figure 2**) and fundus screening (**Figures 3A-D**) on the patient. The results of OCT examination showed that the retinal pigment epithelium (RPE) layer in the macular area of the right eye was disordered and uneven, and irregular mass uplift was seen. The reflection of the ellipsoid zone and the IS/OS layer was interrupted and discontinuous. The RPE layer on the temporal side of the macular showed localized choroidal depression. The macular morphology was irregular, and the thickness of the macular fovea was significantly thinner (**Figure 2A**). However, the results of OCT of the left eye did not show any significant abnormality (**Figure 2B**). The results of fundus screening showed that the right eye had a round-like lesion in the macular area, about 2.5*3PD in size. The lesion was uneven yellow-white, and the center was dark brown. In conjunction with the OCT findings, a bull’s eye macular lesion was considered.

From the results of the ophthalmologic examination, our patient was not considered for the diagnosis of age-related macular degeneration (AMD), which is characterized by the accumulation of extracellular deposits and the progressive degeneration of photoreceptors and adjacent tissues (9–12). The prevalence increases gradually with age and usually affects vision for a short period of time, even leading to blindness (13, 14). In AMD, the damage is concentrated in the central portion of the retina, known as the macula. One of the features of AMD is the scattered or confluent areas of degeneration of RPE cells and overlying photoreceptors in the photoreceptors of the photoreceptors, which depend on the RPE for trophic support. Although our patient’s OCT results showed disturbed and uneven RPE reflection seen in the retina of the macular area of the right eye. However, there was no significant abnormality in the left eye OCT results. Another feature of AMD is the formation of choroidal neovascularization, in which immature blood vessels grow from the choroid below toward the outer retina. These immature blood vessels leak fluid below or inside the retina (10). In contrast, our patient’s funduscopic findings showed no vasculopathy. So we consider that this macular lesion is not so much related to aging as it is to the use of HCQ.

**Figure 4:**
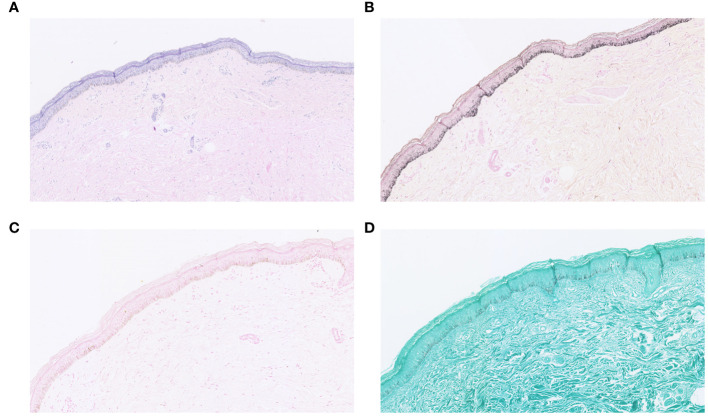
**(A)** HE staining; **(B)** Fontana-Masson staining; **(C)** Prussian blue staining; **(D)** silver hexamine staining.

**Figure 5:**
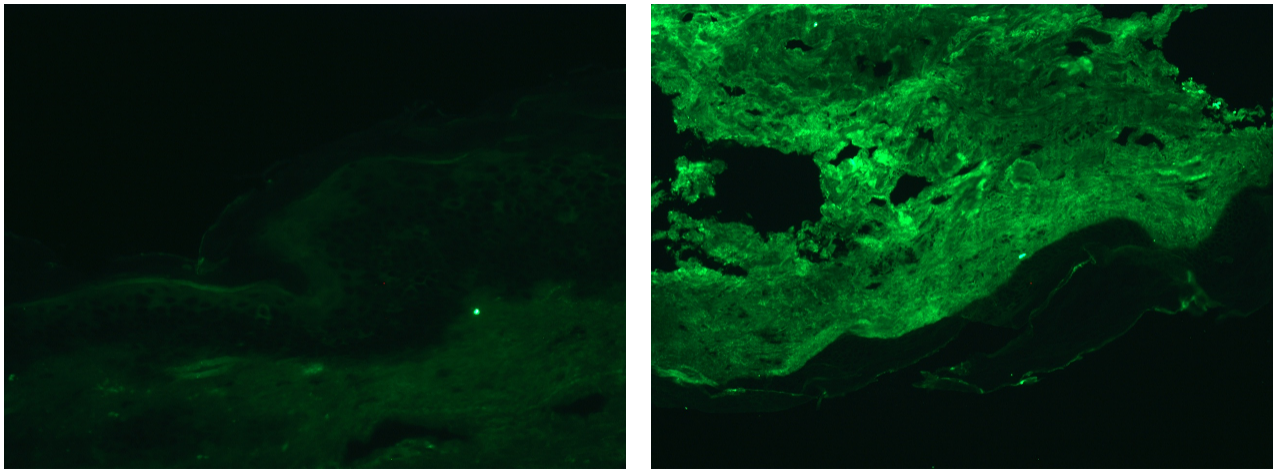
Direct immunofluorescence staining.

In addition, we performed a skin biopsy on the patient. HE staining (**Figure 4A**) showed excessive keratinization of the epidermal mesh basket, a significant increase in melanin in the basal layer, sparse lymphocyte and tissue cell infiltration around the blood vessels in the superficial dermis, and a few melanocytes and melanin granules were seen locally, considering drug-induced pigmentation. Therefore, it can be differentiated from skin pigmentation caused by exposure to ultraviolet light (15). Fontana-Masson staining (**Figure 4B**) showed a significant increase in melanin in the basal layer of the epidermis and a few melanin granules in the superficial dermis. Prussian blue staining and silver hexamine staining were negative (**Figures 4C, D**). Five items of direct immunofluorescence (**Figure 5**) C3, IgG, IgM, IgA and Fib were all negative.

According to the patient ‘s medical history and examination results, we believe that the patient ‘s long-term use of HCQ in the treatment of RA, resulting in hyperpigmentation of the skin and bull’s-eye maculopathy. Therefore, we decided to discontinue HCQ and replace it with iguratimod tablets (50mg/d) and prednisone acetate tablets (5mg/d) to control the disease. It is important to note that iguratimod is only used in China and Japan for the treatment of RA and has been shown to be effective (16–18). Since then, we followed up the patient for three months. After discontinuation of HCQ and treatment with iguratimod tablets, the hyperpigmentation of the patient ‘s skin was gradually reduced, and the symptoms of blurred vision were not significantly improved.

## Discussion

### Literature retrieval

Based on the patient’s clinical presentation and ophthalmologic findings, we reviewed similar cases reported in PubMed, Embase, Web of Science and other databases from the establishment of the database to December 2023. We used “hydroxychloroquine”, “plaquenil”, “case study”, “case report”, “bull’s-eye maculopathy” and “hyperpigmentation” as the search terms to filter out the eligible literature and extract the relevant information of the cases. From the results of our literature search, a total of 181 articles were retrieved from the literature related to hyperpigmentation of the skin. After screening, 18 articles were obtained, the total number of cases was 20, female patients were more than male patients, distributed in different countries or regions, the primary disease was mainly RA or SLE, the color of skin pigmentation was mainly blue/gray, and the site of skin pigmentation was mainly on the face, the upper limbs or the lower limbs, and the daily dosage of HCQ was mainly 400mg, and the skin pigmentation of the over-pigmented skin of the majority of the patients could be gradually subsided after stopping the HCQ. **Table 1** shows the detailed clinical features of these cases. A total of 46 articles were retrieved from the literature related to macular degeneration of the bull’s eye. After screening 11 valid literature were obtained, the total number of cases was 13, all were female patients, the daily dose of HCQ was 200-1200mg and the approximate cumulative dose was 438-2920g, and most of the patients’ solution was to stop HCQ therapy. **Table 2** shows the detailed clinical features of these cases.

**Table 1:** Reported cases of hyperpigmentation of HCQ.

Year	Country	Study	Sample size	Age/Sex	Primary disease	Body parts of hyperpigmentation	Dosage, mg/d	Delay to onset of symptoms/months	Symptoms after discontinuation of HCQ
2002	USA	Ture et al. (19)	1	Not reported	RA	(Black/gray) extremity, torso, hairline	Not reported	Not reported	Not reported
2004	UK	Millard et al. (20)	1	48/F	Mixed connective tissue disease	(Gray)face, neck, trunk, axillae, posterior thighs	400	12	Not reported
2006	UK	Reynaert et al. (21)	2	-/F	SLE	(Blue/gray) pretibial, face	200-400	18	Not reported
2007	Israel	Amichai et al. (22)	1	37/F	RA	(Blue/gray) thighs	400	12	Pigmentation decreased
2008	Turkey	Melikoglu et al. (23)	1	48/F	SS	(Blue/gray) dorsal hands	400	24	Pigmentation decreased
2008	USA	Puri et al. (24)	2	50/F 78/F	Undifferentiated arthritis; SLE and RA	(Gray)upper back, shoulders; (blue)temple	400	48-60/18	Not reported
2008	Netherlands	Rood et al. (25)	1	92/F	RA	(Gray)forearms	400	68	Not reported
2009	USA	Morrison et al. (26)	1	Not reported	SLE	(Blue/gray) shins, forearms, hands	Not reported	Not reported	Not reported
2012	Korea	Cho et al. (27)	1	58/F	RA	(Blue/gray) neck, upper trunk, upper extremities	200	48	Not reported
2013	USA	Mir et al. (28)	1	57/F	SLE	(Blue/gray) face, upper back, feet	Not reported	84	Not reported
2013	USA	Cohen et al. (29)	1	66/F	SLE	(Black)forehead, face, neck, v-area of the upper central chest; (blue)upper right chest	400	396	Not reported
2013	Germany	Tracy et al. (30)	1	48/F	SLE	(Gray)left forehead, nasal bridge, chest, upper back	400	216	Pigmentation decreased
2015	USA	Sawalha et al. (31)	1	32/F	RA	(Black/gray) neck, bilateral forearms, dorsal feet	400	3	Pigmentation decreased
2017	France	Coulombe et al. (32)	1	48/M	SLE	(Blue/gray) face, arms, legs	Not reported	240	Not reported
2018	Portugal	Ivo et al. (33)	1	80/F	SLE and SS	(Brown to grey/black) face, anterior side of legs, forearms	400	132	Not reported
2018	India	Thakur et al. (34)	1	-/F	Granuloma annulare	(Blue/gray) extremities, upper back, neck	300	4	Not reported
2018	Greece	Tosios et al. (35)	1	53/F	RA	(Blue/gray) neck, thorax	400	60	Pigmentation decreased
2018	Turkey	Tekgöz et al. (36)	1	60/M	RA	(Brown/black) face, neck	400	12	Pigmentation decreased

F, female; M, male; RA, rheumatoid arthritis; SLE, systemic lupus erythematosus; SS, Sjögren’s syndrome.

**Table 2:** Reported Adverse Ocular Region Effects of HCQ.

Year	Study	Number of cases	Age/Sex	Dosage(mg/d)	Approximate cumulative dose (g)	The duration of HCQ use(years)	Types of adverse reactions	Resolution
1967	Shearer et al. (37)	1	37/F	600-1200	770	2.21	Visual loss and bilateral bull’s-eye macular degeneration	cessation of HCQ
1987	Johnson et al. (38)	1	43/F	500	730	4	Bilateral pericentral scotomata with a bull’s-eye maculopathy	cessation of HCQ
1991	Weiner et al. (39)	2	49/F, 60/F	400-800, 400	1788, 2920	10, 20	Bull’s-eye maculopathy, window defect was evident by fluorescein angiography in both eyes	cessation of HCQ
2007	Fung et al. (40)	1	48/F	400	1168	8	Bull’s-eye maculopathy	Not reported
2010	Salu et al. (41)	1	43/F	600	657	3	Early stage of bull’s-eye maculopathy	cessation of HCQ
2014	Phillips et al. (42)	1	56/F	400	584	4	Bilateral bull’s-eye macular lesions	Not reported
2015	Wong et al. (43)	2	68/F	Not reported	1168	8	Bull’s-eye maculopathy	Not reported
2016	Brandao et al. (44)	1	15/F	200	438	3	Bull’s-eye maculopathy	cessation of HCQ
2019	Modi et al. (45)	1	60/F	400	2044	14	Bull’s-eye maculopathy	cessation of HCQ
2020	Pellerano et al. (46)	1	28/F	Not reported	1369	15	Bull’s-eye maculopathy	cessation of HCQ
2022	Ameen Ismail et al. (47)	1	42/F	Not reported	Not reported	1.33	Macular degeneration of the left bull’s eye	Not reported

F, female; HCQ, hydroxychloroquine.

### Hyperpigmentation of skin induced by HCQ

Hyperpigmentation of the skin caused by antimalarial treatment has been reported since the Second World War (48). However, the associated skin hyperpigmentation due to HCQ seems to be uncommon compared to other antimalarials such as chloroquine (28, 49). One study showed that the onset of HCQ-associated skin hyperpigmentation ranged from 3 months to 22 years after the start of treatment, with a median of 6.1 years (50). In our reported case, the patient developed hyperpigmentation of the skin about 3 years after the start of treatment, and the treatment was to discontinue HCQs and to control the RA with iguratimod tablets, which led to a gradual reduction of the patient’s skin hyperpigmentation over several months (22, 25). Although there is evidence that both melanin and iron deposition can be present in the dermis in HCQ-induced hyperpigmented skin lesions (50), the exact mechanism is unknown (30). Our patients completed the relevant pathological examination. Fontana-Masson staining (**Figure 4B**) showed a significant increase in melanin in the basal layer of the epidermis and a few melanin granules in the superficial layer of the dermis. Prussian blue staining was negative (**Figure 4C**). Therefore, we describe the pathological features of some cases (**Table 3**). In terms of treatment, most of the investigators took the approach of discontinuing HCQ treatment to prevent further exacerbation of HCQ-induced hyperpigmentation of the skin. Most patients were able to reduce their hyperpigmentation after discontinuing HCQ treatment.

**Table 3:** Pathological features of some cases.

Year	Study	Skin biopsy	Staining
2006	Reynaert et al. (21)	Granular pigment deposition	Not reported
2007	Amichai et al. (22)	Basal hyperpigmentation	Not reported
2008	Puri et al. (24)	Granular pigment deposition	Perls Prussian staining was negative; Fontana-Masson staining was positive
2008	Rood et al. (25)	Basal epidermal hyperpigmentation	Schmorl staining was positive; Iron staining was positive
2012	Cho et al. (27)	Epidermal melanin pigment and superficial dermal, yellow to brown colored granular pigment depositions	Not reported
2013	Mir et al. (28)	Yellow brown, non-refractile and coarsely granular pigment deposits	Fontana-Masson staining was positive
2013	Cohen et al. (29)	Yellow-to-brown granules scattered throughout the reticular dermis	Perls Prussian staining was positive; Fontana-Masson staining was positive
2013	Tracy et al. (30)	Demonstrated pigment within macrophages and within dermal dendrocytes	Perls Prussian staining was positive; Fontana-Masson staining was positive
2017	Coulombe et al. (32)	Yellow brown, nonrefractile and coarsely granular pigment deposition	Perls Prussian staining was positive; Fontana-Masson staining was positive
2018	Ivo et al. (33)	Pigment deposition in superficial dermis	Perls Prussian staining was positive; Fontana-Masson staining was negative
2018	Thakur et al. (34)	Brown coarse pigment in the dermis	Perls Prussian staining was negative
2018	Tekgöz et al. (36)	Accumulation of pigment granules within the collagen fibers and macrophages along with the granules present freely in the tissue	Not reported

### Retinal toxicity due to HCQ

Long-term treatment with HCQ can lead to retinal toxicity (51). This retinal change is typically characterized by a thinning of the photoreceptor layer, starting with a paracentral sulcus ring and progressing over time to a “bull’s-eye” maculopathy, caused by pericentral atrophy and retention of the central sulcus (52). Some researchers have suggested that HCQ may be associated with bilateral retinopathy involving not only the macula but also the peripheral retina (39). The relationship between HCQ-related skin pigmentation and ocular toxicity is currently unknown (28); However, there is a need for regular ophthalmological follow-ups so that the patient’s eye condition can be understood (30). Our patient developed vision loss after 36 months of HCQ treatment, with a cumulative dose of about 438 g. Ophthalmological examination revealed macular lesions in the right eye. **Table 2** describes some of the published information on similar cases, and the case we reported is similar to those reported in the literature. In terms of treatment, most of the investigators took to discontinuing HCQ treatment to prevent further worsening of the ocular adverse effects caused by HCQ. However, patients who have developed bull’s-eye macular degeneration are difficult to rehabilitate (41). In terms of ophthalmic examination, Fung et al. (40) suggest that OCT may be a useful, non-invasive clinical assessment if a patient presents with new visual changes associated with HCQ. In addition, OCT findings of diffuse retinal atrophy or increased reflectivity around the macular central pucker can support the port suspected diagnosis of HCQ-associated retinal toxicity.

## Conclusion

In conclusion, HCQ can cause adverse reactions such as hyperpigmentation of the skin and macular degeneration of the bull’s eye, clinicians should be aware of early cutaneous symptoms and HCQ-associated ophthalmotoxicity in patients with rheumatic diseases on HCQ sulphate and should actively monitor patients, have them undergo regular ophthalmological examinations and give appropriate treatment to prevent exacerbation of symptoms.

## Data availability statement

The original contributions presented in the study are included in the article/supplementary material. Further inquiries can be directed to the corresponding author.

## Ethics statement

The studies involving humans were approved by Ethics Committee of the Second Affiliated Hospital of Guizhou University of Traditional Chinese Medicine. The studies were conducted in accordance with the local legislation and institutional requirements. The participants provided their written informed consent to participate in this study. Written informed consent was obtained from the individual(s) for the publication of any potentially identifiable images or data included in this article.

## Author contributions

J-PP: Writing – original draft, Writing – review & editing. X-YY: Writing – original draft. FL: Writing – original draft. X-MY: Writing – original draft. HX: Writing – original draft. W-KM: Writing – original draft. X-MY: Writing – original draft, Writing – review & editing.
